# Isolated Laryngeal Leishmaniasis in Immunocompetent Patients: An Underdiagnosed Disease

**DOI:** 10.1155/2013/165409

**Published:** 2013-04-15

**Authors:** Salvatore Cocuzza, Alessio Strazzulla, Marilia Rita Pinzone, Stefano Cosentino, Agostino Serra, Rosario Caltabiano, Salvatore Lanzafame, Bruno Cacopardo, Giuseppe Nunnari

**Affiliations:** ^1^Department of Medical-Surgical Specialties, A.O.U. Policlinico-Vittorio Emanuele, University of Catania, 95100 Catania, Italy; ^2^Division of Infectious Diseases, Department of Clinical and Molecular Biomedicine, Garibaldi Nesima Hospital, University of Catania, 95125 Catania, Italy; ^3^Department G.F. Ingrassia, Section of Anatomic Pathology, University of Catania, 95100 Catania, Italy; ^4^Department of Microbiology and Immunology, Jefferson Medical College, Thomas Jefferson University, Philadelphia, PA 19115, USA

## Abstract

We describe a case of isolated primary laryngeal leishmaniasis in an immunocompetent Italian patient with a previous medical history negative for visceral or cutaneous leishmaniasis, presenting with hoarseness. We also summarize the epidemiological, clinical, and diagnostic features and the therapeutic management of other cases of laryngeal leishmaniasis in immunocompetent subjects, described in the literature. Considering the insidious and nonspecific clinical presentation, the increasing number of different forms of mild or underestimated immunosuppressive conditions, and the number of people travelling in endemic zones, along with the ability of *Leishmania* amastigotes to survive for a long period in the body, we believe it is important for pathologists and clinicians to be aware of this unusual form of leishmaniasis in order to avoid delayed recognition and treatment. The rarity of the presentation and the lack of guidelines on mucosal leishmaniasis may contribute to the potential undiagnosed cases or delayed diagnosis, the possible relapses, as well as the correct pharmacological and/or surgical therapeutic approach.

## 1. Introduction

Leishmaniasis is a zoonosis caused by protozoa of the genus *Leishmania* [1] and normally transmitted by the bite of the female *Phlebotomus* (and *Lutzomiya*) sandfly [2]. Extent and localization of lesions depend both on parasite characteristics and host immune response. Leishmaniasis can be clinically classified into three forms: cutaneous leishmaniasis (CL), visceral leishmaniasis (VL), and mucosal/mucocutaneous leishmaniasis (ML) [3].

In leishmaniasis, larynx is more often involved during ML, when mucosal lesions, firstly localized in the nose and/or oral cavity, may progressively descend the upper respiratory tract and sometimes involve the laryngeal mucosa. This is typical of *Leishmania* (L) *braziliensis* infection, which is common in South America [4]. However, some cases of primary laryngeal leishmaniasis, without previous or contemporary involvement of other sites, have been reported. These reports have been associated with *L*.* donovani* and *L*.* infantum* infection, which are typical of the Mediterranean Basin, India, and Sub-Saharan Africa [2].

Here, we present a case of primary isolated laryngeal leishmaniasis in an immunocompetent Italian patient and a review of the literature.

## 2. Case Presentation

In July 2010, a 64-year-old Italian man presented to the Division of Infectious Diseases of the Garibaldi-Nesima Hospital of Catania with an 8-month history of hoarseness and discomfort. He lived in Sicily and he had not travelled outside Europe. His medical history was notable for chronic obstructive pulmonary disease (COPD), diagnosed 20 years before but never treated with corticosteroids, and hypertension. He also referred hypersensitivity to some unspecified nonsteroidal anti-inflammatory drugs and to be a former smoker (25 cigarettes/day). 

On physical examination, no skin lesions were noted; there was neither hepatosplenomegaly nor lymphadenopathy. Laboratory tests were unremarkable. Results of HIV serological tests were negative.

**Figure 1:**
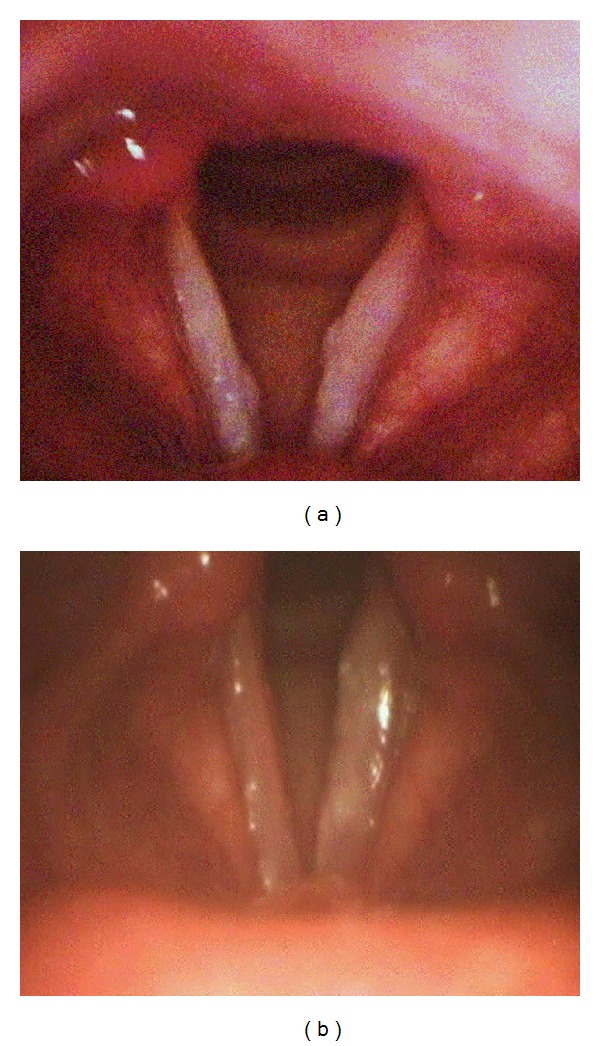
(a) Laryngoscopy showing the presence of two focal hard and whitish lesions of the true vocal cords; (b) significant improvement of vocal cord lesions after treatment with liposomal amphotericine B.

**Figure 2:**
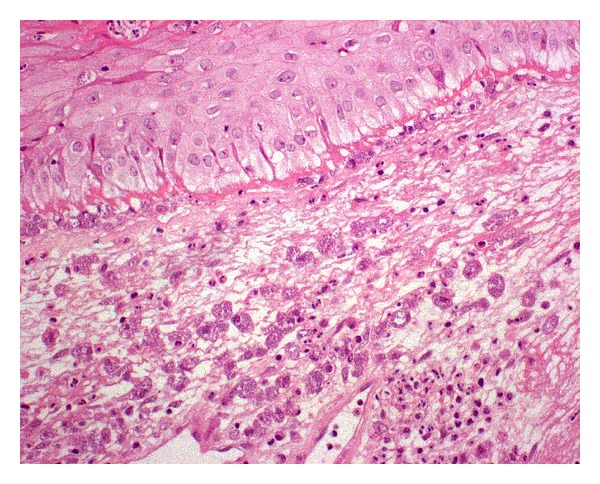
Histological examination of a laryngeal bioptic specimen, showing the presence of *Leishmania* spp. amastigotes in histiocytes (Giemsa ×400)

 In December 2009, before coming to our institute, the patient had already undergone fibroscopy and a laryngeal biopsy because of his symptoms. Histological examination had revealed mucosal inflammation and hyperplasia. On the basis of these results, he had been treated with amoxicillin. In July 2010, because of lack of clinical improvement, a second laryngeal biopsy had been performed. The samples were taken from both vocal cords. Laryngostroboscopic examination showed two focal hard and whitish lesions of the true vocal cords, which had marked attitude to hyperadduction and defect of mucosal wave (Figure 1(a)). Histological examination showed the presence of chronic inflammatory tissue with a wide amount of histiocytes, containing a large number of round parasites, which were referable to amastigotes belonging to *Leishmania *spp. (Figure 2). The results of polymerase chain reaction (PCR) analysis were positive for *L. donovani*. Finally, the patient was treated with liposomal amphotericine B at a dose of 3 mg/kg/per day for 7 days, after which he continued liposomal amphotericine B (3 mg/kg once a week) for 5 weeks. At the end of the therapy, the patient reached complete recovery. 

In June 2011, the patient was readmitted to our institution because of increasing dysphonia. A laryngeal biopsy, done in May 2011, still showed the presence of intracellular *Leishmania* amastigotes, as such the patient was treated with a liposomal amphotericine B-based regimen, at a dose of 3 mg/kg/per day for seven days and, subsequently, 3 mg/kg once a week for 5 weeks. At the end of this treatment, the patient had resolution of dysphonia and, at present, he is in good health. Repeat laryngoscopy showed a significant improvement of vocal cord lesions (Figure 1(b)). 

## 3. Review And Discussion

### 3.1. Case Definition

We report a case of isolated laryngeal leishmaniasis in an immunocompetent patient, who presented to our Infectious Diseases Unit. We also review 15 previously reported cases from 14 articles (Table 1). Other articles were discarded because they did not meet the following criteria: (1) full description of the case; (2) absence of any other lesion related to *Leishmania* infection; (3) absence of local or systemic immunodeficiency; (4) access to English full text or English abstract. Patients were considered immunocompromised in presence of known immune compromising factors, such as viral infections, corticosteroid therapy, or tumors.

## 4. Review of Published Works and Discussion

Sixteen cases [5–18] of laryngeal leishmaniasis are reviewed, including the present report and fifteen previously published cases. The median patient age was 53.5 years (interquartile range 41.5–64 years), and 94% were men. Seven patients (45%) lived in Italy, three (19%) in France, two (12%) in Spain, one (6%) in India, Pakistan, United Kingdom, and Denmark, respectively. Three patients (19%) had a travel history, respectively, around Europe, Spain, and France.

As for comorbidities, a history of smoke was reported in six cases (37.5%); three patients (19%), including the one in the present report, had COPD, one (6%) had type 2 diabetes mellitus, another patient (6%) had thyroid nodules. The patient described here also had hypertension. 

In eleven cases (69%) laryngeal lesions were localized in vocal cords, in two subjects with a consensual involvement of the subglottic region (12%), and in two others of arytenoid cartilage (12%). Subglottic region was the exclusive localization in a case (6%), as well as epiglottis (6%). In three cases (19%) the exact site was not specified.

The most common symptom (8/16 patients, 50%) was dysphonia, isolated (37.5%) or accompanied by dyspnea (37.5%) or dysphagia (25%). Six subjects (37.5%) complained of hoarseness, in one case associated with dyspnea and cough, in another with dysphagia. Less common symptoms (6% in each case) were odynophagia and weight loss. When reported, duration of symptoms ranged between two and eight months.

Histological exam was successful in all cases. Giemsa stain was performed in six cases (37.5%), haematoxylin and eosin staining in four cases (25%). Antileishmania antibodies were positive in six cases (37.5%).

In differential diagnosis, neoplasia was considered in five cases (31%), histoplasmosis in two cases (12%); tuberculosis, toxoplasmosis, and unspecific infection were each considered in one case (6%).

Identification of *Leishmania* spp. was made in seven cases (45%): L *infantum* was identified in three cases, by culture and isoenzyme analysis or PCR, in four others L. *donovani *was isolated by PCR or immunoperoxidase analysis. In a case, a generic diagnosis of infection by *Leishmania* spp. was made by PCR. 

In seven cases (45%), therapy was performed with liposomal amphotericine B, in five cases (31%) with meglumine antimoniate, and in two cases (12%) with surgery. After treatment, twelve patients (75%) clinically recovered and one (6%) only partially recovered.

Isolated laryngeal leishmaniasis has been related with *L. donovani* and *L. infantum* infection [2, 19, 20]. Indeed, to our knowledge cases of isolated laryngeal leishmaniasis associated with *L. braziliensis *infection have not been reported so far. It could mean that only some strains belonging to *L. donovani* complex are able to adapt themselves to electively live in the laryngeal tissue. Otherwise, the final site of lesions may be determined by the effectiveness of the host immune response against *Leishmania.* In this case, laryngeal lesions would mirror the capability of macrophages to confine the disease. 

Leishmaniasis is transmitted by sandfly bite [1], but it appears unlikely to hypothesize a direct injection of protozoa in the laryngeal mucosa. More probably, *Leishmania* (free or carried by macrophagic cells) may reach the laryngeal tissue by lymphatics and bloodstream, after being inoculated in more accessible sites. Unfortunately, the site of injection is generally undetectable.

The localization of *Leishmania* in the laryngeal mucosa is promoted by local or systemic immunodeficiency, for example, HIV infection [21] and corticosteroid therapy [22]. In immunocompetent patients, the absence of any documented immunodeficiency, such as in our case report and reviewed cases, suggests the need to speculate about other risk factors. Aliaga and colleagues supposed that the lower temperature of the upper aerodigestive tract might help the survival of adapted *Leishmania* strains [3]. Inflammation of the upper respiratory tract, associated with smoke [8, 11, 16–18] or chronic respiratory diseases [14, 18], could also play an important role. Infected macrophages may localize to the larynx as a result of local constant inflammation.

In isolated laryngeal leishmaniasis, laryngeal lesions are not specific, as well as symptoms. In fact, they may mimic many inflammatory and neoplastic diseases. Diagnosis is usually further complicated by the absence of any documented history of CL. Furthermore, comorbidities are often misleading for physicians because, at first glance, symptoms may be easily referred to comorbidities, such as in the case described by Guddo and colleagues, who attributed patient's symptoms to COPD [14]. As a consequence, also considering the low incidence of this atypical localization, laryngeal leishmaniasis is hardly evaluated in differential diagnosis by physicians. However, the spread of *Leishmania* vectors in nonendemic zones [19] and the increasing number of people travelling in endemic zones suggest to suspect leishmaniasis as a possible explanation for laryngeal symptoms. Moreover, because of the ability of protozoal amastigotes to survive for a long time in human body [1, 4], leishmaniasis has to be suspected even if the patient had visited or lived in endemic zones many years before. Grant and colleagues described a case of isolated mucosal leishmaniasis diagnosed sixteen years after the probable infection [23].

Suspected leishmaniasis needs laboratory tests to be confirmed. Histological evaluation of biopsies is usually able to confirm diagnosis [20]. Nevertheless, our case report shows that it sometimes fails to detect *Leishmania* amastigotes. Probably, before performing a histological examination, the physician should inform the pathologist about his suspicion in order to help him in identifying protozoa. Other exams, like leishmanin skin test (LST) and the detection of antileishmania antibodies, can only support diagnosis. Instead, PCR is a highly sensitive and specific molecular method in detecting the presence of *Leishmania* DNA and identifying the species. Unfortunately, PCR is not always available [20].

Pentavalent antimonials (sodium stibogluconate and meglumine antimoniate) and liposomal amphotericine B are the most used drugs for leishmaniasis; for VL, their recommended doses are, respectively, 20 mg/kg/day for 28 days and 3–5 mg/kg/day over a 3–6 days period, up to a total dose of 18–21 mg/kg [24]. Unfortunately, little evidence is available for the treatment of laryngeal leishmaniasis, because of the limited number of reported cases, which makes it difficult to establish the most appropriate therapeutic approach. The role of surgery for laryngeal leishmaniasis is currently undefined, because, again, available evidence does not allow to evaluate if the surgical option (alone or together with medical treatment) may adequately work for laryngeal leishmaniasis.

Timing of followup is another controversial point; all but one of the cases were characterized by clinical recovery and no relapses, but timing of followup was not always reported, and it is unclear if the absence of relapses 8–12 months after treatment is enough to rule out the risk of reactivation. In fact, after treatment for leishmaniasis, relapses may occur [4]. In two cases of isolated laryngeal leishmaniasis, immunosuppressed patients developed VL after successful treatment [12, 21]. Our case report shows that relapses are possible also in immunocompetent patients maybe because of the inability of the immune system to effectively control infection and eliminate protozoa.

## 5. Conclusion

There are some important clinical lessons that can be drawn from the existing case literature and are reinforced by our case report; the first message is that, even if rare, primary isolated laryngeal leishmaniasis may occur in immunocompetent subjects, even in the absence of previous visceral, cutaneous, or mucocutaneous leishmaniasis, and represent an underestimated cause of laryngeal symptoms. Physicians should include leishmaniasis in their diagnostic schedule, especially if the patient has lived or travelled in endemic zones. Early diagnosis is crucial, as leishmaniasis is a treatable disease, whose natural history may be heavily changed by proper recognition and treatment. PCR is the gold standard to confirm the presence of protozoa but, unfortunately, it is not routinely available. Accessibility to PCR testing should be implemented, because it represents the most sensitive and specific diagnostic tool in our hands. 

Considering the insidious and nonspecific clinical presentation, the increasing number of different forms of immunosuppression, and the number of people travelling in endemic zones, along with the ability of *Leishmania* amastigotes to survive for a long period in the body, we believe it is important for physicians to consider leishmaniasis as a possible cause of laryngeal symptoms, even in immunocompetent subjects. 

Finally, more research is needed to shed some light on the mechanisms behind immunity to *Leishmania* in order to explain how laryngeal leishmaniasis may occur even in the absence of evident immune compromising risk factors. More importantly, a therapeutic algorithm, pharmacological, surgical, or combined, has never been investigated, as well as the first or second line regimen, doses, and duration of treatment. Furthermore, the possibility of relapses has to be kept in consideration.

## Figures and Tables

**Table 1:** Features of 16 cases of isolated laryngeal leishmaniasis in immunocompetent subjects.

Reference	Age	Sex	Nation	Comorbidities	Lesion site	Lesion description	Signs and Symptoms	Diagnosis	Differential diagnosis	*Leishmania * spp.	Therapy	Outcome
[5]	70	M	India	Thyroid nodules	Subglottic region and vocal cords	Pinkish-white mass	Hoarseness, dyspnea, cough, and noisy breathing	Histological	—	Unknown, probably *L*. *donovani *	Liposomal amphotericine B daily for 14 days	Clinical recovery, no relapse after 8 months

[6]	53	M	Italy	None	Right epiglottis and pharyngo-laryngeal wall	Whitish fungating region	Dysphonia, dyspnea, and odynophagia	Histological (Giemsa)	Histoplasmosis	*L*. *infantum* (PCR)	Liposomal amphotericine B (3 mg/kg/day), for 5 days, repeated after 10 days	Clinical recovery, no relapse after a year

[7]	30	F	Pakistan	None	Right vocal cord	Ulcerative nodular mass	Dysphonia and difficult breathing	Histological	Neoplasia	—	—	—

[8]	64	M	Italy	Diabetes, previous heavy smoker	Left vocal cord	—	Dysphonia	Histological antileishmania antibodies	Neoplasia	—	Liposomal amphotericine B (0.5 mg/kg/day)	Partial clinical recovery

[9]	35	M	Italy	None	Vocal cords	Ulcer	Dysphonia, dysphagia	Histological	—	—	Meglumine antimoniate (0.1 g/day) for 3 days	Clinical recovery

[10]	54	M	Italy	None	Larynx	Swelling	Dysphonia, dysphagia	Histological (Giemsa)	—	—	Meglumine antimoniate for 20 days	Clinical recovery

[11]	42	M	Italy	Smoker	Right vocal cord	Polypoid lesion	Hoarseness	Histological (Giemsa)	—	*L*. *donovani *(immunoperoxidase)	Microsurgery	Clinical recovery, no relapse after a year

[12]	49	M	France	None	Vocal cords	Nodular lesion	Dysphonia	Histological antileishmania antibodies	—	—	Meglumine antimoniate (850 mg/day) for 21 days	—

[12]	40	M	France	None	Vocal cords	Ulcerative lesion	—	Histological antileishmania antibodies	—	—	Amphotericine B (3 mg/kg/day) at day 1, 2, 3, 4, 5, 10	Clinical recovery

[13]	78	M	Denmark	—	Larynx	—	Hoarseness	Histological antileishmania antibodies	—	*L*. *donovani* or *L*. *infantum* or *L*. *tropica* (PCR)	—	—

[14]	59	M	Italy	COPD	Subglottic region	Polypoid lesion and erythema	Cough, mucus production	Histological (Giemsa) antileishmania antibodies	Toxoplasmosis Histoplasmosis	*L*. *donovani* (PCR)	Liposomal amphotericine B (0.5 mg/kg/day) for 10 days	Clinical recovery

[15]	56	M	France	None	Larynx	Polypoid lesion	Hoarseness	Histological antileishmania antibodies	—	—	Surgery	Clinical recovery

[16]	36	M	Spain	Smoker	Left vocal cord, arytenoid cartilage, and epiglottis	Tumor-like lesion	Dysphonia	Histological (haematoxylin and eosin, Giemsa )	Bacterial, fungal, and mycobacterial infections	*L*. *infantum* (culture and isoenzyme analysis)	Meglumine antimoniate (850 mg/day) for 28 days	Clinical recovery, no relapse after 3 months

[17]	49	M	Spain	Smoker, alcohol drinker	Left vocal cord and subglottic region	Vegetant ulcerated lesion	Dysphonia, dyspnea, and weight loss	Histological (haematoxylin and eosin)	Tuberculosis neoplasia	—	Meglumine antimoniate (10 mg/day) for 2 months	Clinical recovery

[18]	84	M	United Kingdom	COPD, former smoker	Left vocal cord	Inflammation	Hoarseness and dysphagia	Histological (haematoxylin and eosin, Giemsa)	Neoplasia	*L*. *donovani* (PCR)	Liposomal amphotericin B (3 mg/kg/day) at day 1, 2, 3, 4, 5, 14, and 21	Clinical recovery

Reported here	64	M	Italy	COPD, hypertension, former smoker	Vocal cords	Inflammation and mucosal hyperplasia	Hoarseness and discomfort	Histological (haematoxylin and eosin )	Neoplasia	*L*. *infantum* (PCR)	Liposomal amphotericine B (3 mg/kg/day) for 7 days, then once a week for 5 weeks	Clinical recovery, relapse after 3 months

—: not reported/unknown.

COPD: chronic obstructive pulmonary disease; PCR: polymerase chain reaction.

## References

[1] David CV, Craft N (2009). Cutaneous and mucocutaneous leishmaniasis. *Dermatologic Therapy*.

[2] Piscopo TV, Azzopardi CM (2007). Leishmaniasis. *Postgraduate Medical Journal*.

[3] Aliaga L, Cobo F, Mediavilla JD (2003). Localized mucosal Leishmaniasis due to Leishmania (Leishmania) infantum clinical and microbiologic findings in 31 patients. *Medicine*.

[4] Goto H, Lindoso JAL (2010). Current diagnosis and treatment of cutaneous and mucocutaneous leishmaniasis. *Expert Review of Anti-Infective Therapy*.

[5] Kumar B, Ghimire A, Karki S, Upadhyaya P (2009). Primary laryngeal leishmaniasis: a rare case report. *Indian Journal of Pathology and Microbiology*.

[6] Casolari C, Guaraldi G, Pecorari M (2005). A rare case of localized mucosal leishmaniasis due to Leishmania infantum in an immunocompetent italian host. *European Journal of Epidemiology*.

[7] Iqbal K, Abbasi ZI, Gomal HK (2005). Leishmania of the vocal cords: an unusual site. *Journal of Medical Sciences*.

[8] Tiseo D, Tosone G, Conte MCD (2008). Isolated laryngeal leishmaniasis in an immunocompetent patient: a case report. *Infezioni in Medicina*.

[9] Pototschnig B (1964). Leishmaniasis of the larynx. *L'Oto-Rino-Laringologia Italiana*.

[10] ’Anna M D, Jemma S (1964). On clinically primary laryngeal Leishmaniosis. *Archivii Italiani di Laringologia*.

[11] Ferlito A, Pesavento G, Visonà A, Recher G, Meli S, Bevilacqua P (1986). Leishmaniasis donovani presenting as an isolated lesion in the larynx. *Journal for Oto-Rhino-Laryngology and Its Related Specialties*.

[12] Faucher B, Pomares C, Fourcade S (2011). Mucosal leishmania infantum leishmaniasis: specific pattern in a multicentre survey and historical cases. *Journal of Infection*.

[13] Kaltoft M, Munch-Petersen HR, Møller H (2010). Leishmaniasis isolated to the larynx as cause of chronic laryngitis. *Ugeskrift for Laeger*.

[14] Guddo F, Gallo E, Cillari E (2005). Detection of Leishmania infantum kinetoplast DNA in laryngeal tissue from an immunocompetent patient. *Human Pathology*.

[15] Lanotte G, Rioux JA, Pratlong F (1980). Ecologie des leishmanioses dans le sud de la France. 14. Les leishmanioses humaines en Cevennes. Analyse clinique et biologique des formes viscerales et muqueuses. *Annales de Parasitologie Humaine et Comparée*.

[16] Benítez MD, Miranda C, Navarro JM, Morillas F, Martín J, de la Rosa M (2001). Thirty-six year old male patient with dysphonia refractory to conventional medical treatment. *Enfermedades Infecciosas y Microbiología Clínica*.

[17] Bodet E, Andreu L, Ruiz Giner A, Fortuny JC, Palomar V (2002). Leishmaniasis laríngea: presentación de dos casos clínicos. *Revista Internacional de Otorrinolaringología*.

[18] Teemul TA, Williams J, Lester SE (2012). Laryngeal leishmaniasis: case report of a rare infection. *Head & Neck*.

[19] Ready PD (2010). Leishmaniasis emergence in Europe. *Euro Surveillance*.

[20] Singh S (2006). New developments in diagnosis of leishmaniasis. *Indian Journal of Medical Research*.

[21] Gonzalez-Anglada MI, Pena JM, Barbado FJ (1994). Two cases of laryngeal leishmaniasis in patients infected with HIV. *European Journal of Clinical Microbiology and Infectious Diseases*.

[22] Cánovas DL, Carbonell J, Torres J, Altés J, Buades J (1994). Laryngeal leishmaniasis as initial opportunistic disease in HIV infection. *The Journal of Laryngology & Otology*.

[23] Grant A, Spraggs PDR, Grant HR, Bryceson ADM (1994). Laryngeal leishmaniasis. *Journal of Laryngology and Otology*.

[24] WHO (March 2010). Report of a meeting of the WHO Expert Committee on the Control of Leishmaniases.

